# Expression dynamics of periodic transcripts during cancer cell cycle progression and their correlation with anticancer drug sensitivity

**DOI:** 10.1186/s40779-022-00432-w

**Published:** 2022-12-19

**Authors:** Chun-Xiao Li, Jin-Song Wang, Wen-Na Wang, Dong-Kui Xu, Yan-Tong Zhou, Fang-Zhou Sun, Yi-Qun Li, Feng-Zhu Guo, Jia-Lu Ma, Xue-Yan Zhang, Meng-Jiao Chang, Bing-He Xu, Fei Ma, Hai-Li Qian

**Affiliations:** 1 State Key Laboratory of Molecular Oncology, National Cancer Center/National Clinical Research Center for Cancer/Cancer Hospital, Chinese Academy of Medical Sciences and Peking Union Medical College, Beijing, 100021 China; 2 Department of Medical Oncology, National Cancer Center/National Clinical Research Center for Cancer/Cancer Hospital, Chinese Academy of Medical Sciences and Peking Union Medical College, Beijing, 100021 China; 3 Department of VIP, National Cancer Center/National Clinical Research Center for Cancer/Cancer Hospital, Chinese Academy of Medical Sciences and Peking Union Medical College, Beijing, 100021 China

**Keywords:** Cell cycle, Alternative splicing, Transcriptome, Drug resistance, Cyclin dependent kinase 4/6 inhibitor, Dolichyl-phosphate mannosyltransferase polypeptide 2

## Abstract

**Background:**

The cell cycle is at the center of cellular activities and is orchestrated by complex regulatory mechanisms, among which transcriptional regulation is one of the most important components. Alternative splicing dramatically expands the regulatory network by producing transcript isoforms of genes to exquisitely control the cell cycle. However, the patterns of transcript isoform expression in the cell cycle are unclear. Therapies targeting cell cycle checkpoints are commonly used as anticancer therapies, but none of them have been designed or evaluated at the alternative splicing transcript level. The utility of these transcripts as markers of cell cycle-related drug sensitivity is still unknown, and studies on the expression patterns of cell cycle-targeting drug-related transcripts are also rare.

**Methods:**

To explore alternative splicing patterns during cell cycle progression, we performed sequential transcriptomic assays following cell cycle synchronization in colon cancer HCT116 and breast cancer MDA-MB-231 cell lines, using flow cytometry and reference cell cycle transcripts to confirm the cell cycle phases of samples, and we developed a new algorithm to describe the periodic patterns of transcripts fluctuating during the cell cycle. Genomics of Drug Sensitivity in Cancer (GDSC) drug sensitivity datasets and Cancer Cell Line Encyclopedia (CCLE) transcript datasets were used to assess the correlation of genes and their transcript isoforms with drug sensitivity. We identified transcripts associated with typical drugs targeting cell cycle by determining correlation coefficients. Cytotoxicity assays were used to confirm the effect of ENST00000257904 against cyclin dependent kinase 4/6 (CDK4/6) inhibitors. Finally, alternative splicing transcripts associated with mitotic (M) phase arrest were analyzed using an RNA synthesis inhibition assay and transcriptome analysis.

**Results:**

We established high-resolution transcriptome datasets of synchronized cell cycle samples from colon cancer HCT116 and breast cancer MDA-MB-231 cells. The results of the cell cycle assessment showed that 43,326, 41,578 and 29,244 transcripts were found to be periodically expressed in HeLa, HCT116 and MDA-MB-231 cells, respectively, among which 1280 transcripts showed this expression pattern in all three cancer cell lines. Drug sensitivity assessments showed that a large number of these transcripts displayed a higher correlation with drug sensitivity than their corresponding genes. Cell cycle-related drug screening showed that the level of the CDK4 transcript ENST00000547281 was more significantly associated with the resistance of cells to CDK4/6 inhibitors than the level of the CDK4 reference transcript ENST00000257904. The transcriptional inhibition assay following M phase arrest further confirmed the M-phase-specific expression of the splicing transcripts. Combined with the cell cycle-related drug screening, the results also showed that a set of periodic transcripts, for example, ENST00000314392 (a dolichyl-phosphate mannosyltransferase polypeptide 2 isoform transcript), was more associated with drug sensitivity than the levels of their corresponding gene transcripts.

**Conclusions:**

In summary, we identified a panel of cell cycle-related periodic transcripts and found that the levels of transcripts of drug target genes showed different values for predicting drug sensitivity, providing novel insights into alternative splicing-related drug development and evaluation.

**Supplementary Information:**

The online version contains supplementary material available at 10.1186/s40779-022-00432-w.

## Background

The cell cycle is a critical process in cellular biology and has been the focus of cancer research. The cell cycle consists of four typical phases: G_0_/G_1_, S (DNA synthesis), G_2_, and M (mitosis). These four phases enable exquisite control of DNA replication and cell division cycles and are highly conserved. The main processes during the cell cycle include related cellular functions during each phase, the control of cell cycle checkpoints, replication of cellular components, and mechanisms for the fine control of cell division [1]. Transcriptional regulation is an important mechanism for managing cell cycle transitions. A set of genes periodically expressed throughout the cell cycle has been identified based on their expression patterns and molecular functions [2, 3], most of which are cell cycle checkpoint regulators, which include cyclins and cyclin dependent kinases (CDKs) [4]. However, there are still many questions regarding the precise transcriptional regulation of the cell cycle.

Alternative splicing is a key mechanism that developed across the course of evolution to enable complex cellular functions to be performed in eukaryotic cells. mRNA is the main molecule involved in alternative splicing; pre-mRNA is spliced, and exons are joined selectively together into mature mRNA. Alternative splicing transcripts from a gene vary greatly in their mature sequences, resulting in multiple functional transcript isoforms that can induce various biological activities, greatly maximizing the complexity and controllability of gene function regulation [5]. During cell cycle transitions, almost all transcription processes are suspended, leaving alternative splicing and RNA half-life control as the main ways to regulate the levels of proteins and their functions [6, 7]. The amount of alternatively spliced transcripts that are responsible for cell cycle regulation fluctuates during the cell cycle in a conserved pattern [8]. These periodic alternative splicing transcripts are an essential force that drives or halts cell cycle progression. Understanding cell cycle-related periodically expressed transcripts will facilitate the development of novel solutions for cell cycle-dependent diseases, such as cancer [9].

Cancer is a disease that involves the dysregulation of the cell cycle, which results in uncontrolled cell proliferation and visible tumors [10]. Therapeutic strategies targeting the cell cycle, such as CDK4/6 inhibitors, are important modern tumor therapies; there has been rapid progress in both the study of these strategies and their use in clinical practice [11], although most of the existing strategies have not been developed with alternatively spliced transcript levels in mind. It is of great significance to study the precise alternative splicing pattern during the cell cycle and cell cycle-related transcript functions in cancer progression. Our previous studies indicated that aberrant alternative splicing had significant effects on cancerous phenotypes through cell cycle modulation [12]. Some studies have reported abnormalities in alternative splicing processes in tumors [13–15]. However, few of these studies have revealed the exact transcript fluctuation patterns and their significance in cancer treatment due to the complexities and technical shortcomings.

To further explore the precise alternative splicing patterns during the cancer cell cycle and the relationship of the levels of alternative splicing transcripts with sensitivity to cell cycle-targeted drugs, we combined cell cycle synchronization and high-resolution transcriptome analysis to establish a new algorithm to identify periodic transcripts during the cell cycle. Furthermore, since transcript-based research in clinical settings is rare [16], we integrated Genomics of Drug Sensitivity in Cancer (GDSC) pharmacogenomic datasets and Cancer Cell Line Encyclopedia (CCLE) transcriptomic datasets to explore the value of transcripts in drug sensitivity prediction. These findings will provide significant insights into transcript dynamics during the cancer cell cycle and the significance of transcript patterns in predicting sensitivity to cell cycle-targeted chemotherapeutics, which will greatly enhance anticancer drug research and development.

## Methods

### Cell culture

The HCT116, MDA-MB-231, and MCF7 cell lines were obtained from the National Infrastructure of Cell Line Resources (Beijing, China). All the cell lines were cultured in DMEM plus 10% FBS. All cells were maintained in humidified incubators with 5% CO_2_. The cells were maintained at 30–90% confluence and passaged at 1:2–1:3 ratio after 0.25% trypsin digestion.

### Cell cycle synchronization

Synchronization of the G_1_/S phase of the cell cycle was performed by following the protocol previously described by Dominguez et al. [17]. Cells were plated into 6 cm dishes in complete media at 30% confluence and allowed to attach for 16 h. For the first arrest of the cell cycle, HCT116 cells were treated with 2 mmol/L thymidine for 24 h, washed 3 times with PBS, and then supplemented with fresh complete medium for 10 h; 2 mmol/L thymidine was subsequently added for a second arrest and incubated for 14 h, and then the successful blockade of the cell cycle in G_1_/S phase was confirmed. For MDA-MB-231 cells, the first round of blockade was carried out by treatment with 2 mmol/L thymidine for 22 h, 3 washes with PBS, and then supplementation with fresh complete media for 8 h; in the second round of blockade, the cells were treated with 2 mmol/L thymidine for 18 h, and successful blockade of the cell cycle in G_1_/S phase was confirmed. All the cells were washed 3 times with PBS and cultured in fresh complete medium for cell cycle release. HCT116 cells were harvested at 0, 3, 7, 8, 9, 10, 11 and 13 h after G_1_/S phase release; MDA-MB-231 cells were harvested at 0, 3, 4.5, 6, 7, 9, 10, 11.5, 13, and 16 h after G_1_/S phase release. For M phase arrest, when MDA-MB-231 cells were released from G_1_/S phase, 1 µg/ml nocodazole was added, and after 10 h, they were supplemented with 5 µg/ml actinomycin D.

### Cell cycle analysis

Cells were collected with trypsin digestion, washed with PBS, and fixed in 70% ethanol at − 20 ℃ for at least 4 h. Alcohol was removed by centrifugation. The cells were washed with PBS, PI staining solution was added and incubated for 10 min, and the cells were analyzed by flow cytometry.

### Transcript plasmid construction

To construct the CDK4 transcript vector, the CDSs of ENST00000257904 and ENST00000547281 transcripts were cloned between the BamH1 and Not1 sites of the pCDNA3.1(+) vector.

### Transfection of plasmids

MCF7 cells were transfected with the plasmid vectors using Lipofectamine 2000 reagent (Thermo Fisher, USA) according to the protocol and when the cells reached 70% confluence in 6-well plates. The amount of transfection reagent and plasmid used were 5 μl and 2.5 μg, respectively. Six hours after transfection, cells were seeded into plates for subsequent experiments.

### Cell viability analysis

Cell proliferation assays were performed using the xCELLigence Real-Time Cell Analyzer system (Roche, Switzerland). Cells were seeded in a 16-well E-plate at 30% confluence with 200 μl medium/well, and two replicates on an E-plate were performed. Cytotoxicity experiments were performed by adding ribociclib (MedChemExpress, USA) to the system 14 h after the cells were seeded into the E-plate. Proliferation and drug toxicity assays were repeated 3 times using MTS experiments (Promega, USA) according to the protocol.

### Quantitative real-time PCR

Forty-eight hours after transfection, total RNA was extracted using TRIzol reagent (Invitrogen, USA) according to the manufacturer’s protocol. Total RNA (100 ng) was reverse-transcribed into cDNA with RevertAid reagent (Thermo, USA) using Oligo (dT) 18. Quantification of mRNA expression was performed using ABI QSDX and QuantStudio 5 (Thermo, USA). The primers used were as follows: ENST00000257904 primer: forward: 5′-CAGTTCGTGAGGTGGCTTTA-3′, reverse: 5′-TCCTTAGGTCCTGGTCTACATG-3′; ENST00000547281 primer: forward: 5′-AGGTAACCCTGGTGTTTGAGC-3′, reverse: 5′-AATTGGCATGAAGGAAATCTAG-3′.

### RNA-seq

For HeLa cells, we downloaded the raw RNA-seq data of samples 1 to 8 from GSE81485. For the HCT116 and MDA-MB-231 cell lines, the cells were collected with trypsin digestion and washed with PBS, and RNA was extracted using TRIzol. After strand-specific library construction, Novaseq6000 was used for sequencing, and the raw data of each sample were more than 10 Gb. After all the FASTQ files were quality controlled, BAM files and fragments per kilobase of exon model per million mapped fragments (FPKM) values of transcripts were analyzed with HISAT2 and StringTie, data were mapped with the GRCH38 genome, and the frequency of alternative splicing was calculated with rMATS4.0.1.

### Identification of periodic transcripts

In the RNA-seq FPKM matrix, 8 samples of HeLa or HCT116 cells and 10 samples of MDA-MB-231 cells were ordered according to our cell cycle synchronization sample collection protocol. We generated an algorithm to identify the periodic transcripts. Our assessment of periodic transcripts consisted of two parts. The first part consisted of the following 3 steps: 1) calculate *i* and *n* at the maximum *S*_3_ to determine the position of the periodic peak; 2) for groups of 8 samples in total, use *r*_3_ > 18 to screen for significant peaks, and for groups of 10 samples in total, use *r*_3_ > 24 to screen for significant peaks; 3) use *k*_3_ > 0.3 to avoid collapse in the middle of peaks. The algorithm is as follows:

$$k_{3} = \frac{{a_{i} }}{{max\left( {\mathop \sum \nolimits_{i = n - 1}^{n + 1} a_{i} } \right)}}$$

where *a*_*i*_ is the FPKM value of a transcript in sample *i*. For HeLa and HCT116 cells, *n* = 1 to 8, and *a*_0_ = *a*_8_, *a*_9_ = *a*_1_. For MDA-MB-231 cells, *n* = 1 to 10, and *a*_0_ = *a*_10_, *a*_11_ = *a*_1_. $S_{3} = \mathop \sum \limits_{i = n - 1}^{n + 1} a_{i}$, when *S*_3_ is maximum, for the *n*:

$$r_{3} = \mathop \sum \limits_{i = n - 1}^{n + 1} R_{i}$$

*R*_*i*_ is the value rank of *a*_*i*_ among all samples. For HeLa and HCT116 cells, *i* = 1 to 8, and for MDA-MB-231 cells, *i* = 1 to 10. For the second part of the calculation of the periodic peak, the sample size was increased from 3 to 5, and included the following 4 steps: 1) calculate *i* and *n* at the maximum *S*_5_; 2) use *r*_5_ > 25 for groups of 8 samples in total and *r*_5_ > 35 for groups of 10 samples in total; 3) use *k*_5_ > 0.6; 4) use *k*_3_ > 0.3. The algorithm was as follows:

$$k_{5} = \frac{{a_{i - 1} + a_{i} + a_{i + 1} }}{{max\left( {\mathop \sum \nolimits_{i = n - 2}^{n + 2} a_{i} } \right)}}$$

For HeLa and HCT116 cells, *n* = 1 to 8, and *a*_-1_ = *a*_7_, *a*_0_ = *a*_8_, *a*_9_ = *a*_1_, *a*_10_ = *a*_2_. For MDA-MB-231 cells, *n* = 1 to 10, and *a*_-1_ = *a*_9_, *a*_0_ = *a*_10_, *a*_11_ = *a*_1_, *a*_12_ = *a*_2_. $S_{5} = \mathop \sum \limits_{i = n - 2}^{n + 2} a_{i}$, when *S*_5_ is maximum, for the *n*,

$$k_{3} = \frac{{a_{i} }}{{\mathop \sum \nolimits_{i = n - 1}^{n + 1} a_{i} }}$$

$$r_{5} = \mathop \sum \limits_{i = n - 2}^{{{\text{n}} + 2}} R_{i}$$

### Heatmap and cluster analyses

The periodic transcript FPKM matrix of HeLa, HCT116 and MDA-MB-231 cells were sorted according to the sample number of the periodic peaks, and GENE-E was used to draw the heatmap. The periodic transcripts were subjected to Gene Ontology Biological Process (GO-BP) and Kyoto Encyclopedia of Genes and Genomes (KEGG) enrichment analyses with DAVID [18], and diagrams were drawn with ggplot2.

### Identification of drug sensitivity-related transcript isoforms

**Fig. 1:**
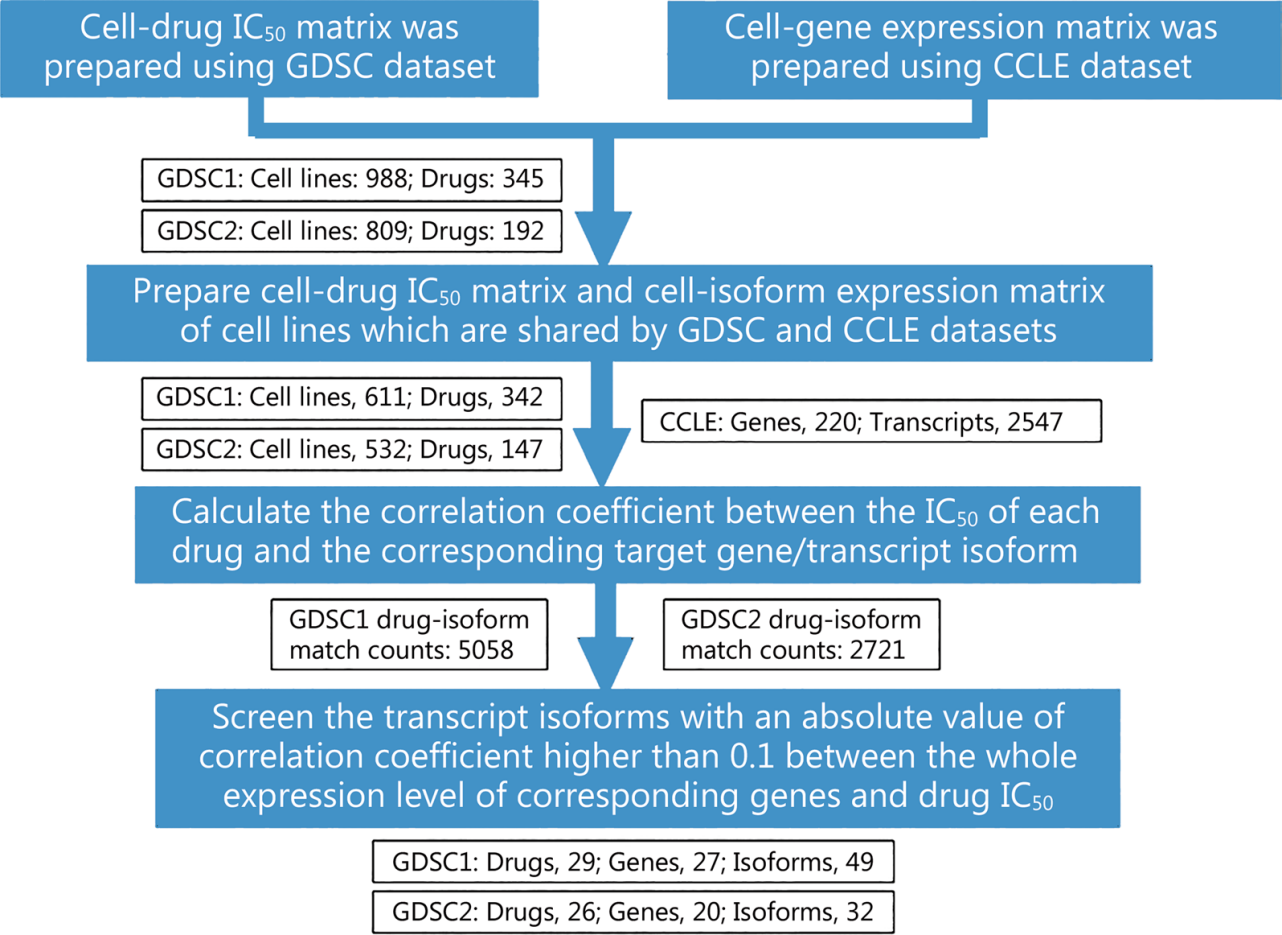
Analysis pipeline of drug sensitivity-related transcripts and the number of drugs, genes, and transcripts identified in each step. The data processing steps (in blue) are used to match the data in GDSC and CCLE datasets and screen drug sensitivity transcripts. The matching and screening number of drugs, genes, and transcripts in each step are shown in the box (in white). GDSC Genomics of Drug Sensitivity in Cancer, CCLE cancer cell line encyclopedia

In this study, the transcript isoforms of genes targeted by drugs were analyzed. To identify the relationship between the transcript isoform level and corresponding drug sensitivity in the cell lines, we developed a pipeline to automatically match drugs and their target gene transcript isoforms and performed Spearman's correlation analyses of the drug 50% inhibitory concentration (IC_50_) values and matched isoform levels (Fig. 1). First, we used GDSC data to establish a cell-drug IC_50_ matrix. We then used CCLE data to establish a cell-gene/isoform expression matrix and input the two matrices into the pipeline. In the pipeline, the drugs and corresponding target genes/transcript isoforms in the two matrices were matched one by one, and we calculated the correlation between drug IC_50_ values and gene/transcript isoform levels in various cell lines. After obtaining the correlation coefficient matrix between all drug target gene/transcript isoform levels and drug IC_50_ values, we compared the correlation coefficients for the individual transcript isoforms or their corresponding genes. The transcript isoforms with an absolute value of correlation coefficient 0.1 higher than their corresponding genes were defined as drug sensitivity-related transcripts.

### Statistical analysis

Survival analysis was performed and plotted using the KMplot database [19]. Venn diagram analysis was performed using http://bioinformatics.psb.ugent.be/webtools/Venn. The continuous variables were presented as the mean ± SD and analyzed by Student’s *t*-test after Shapiro–Wilk and Levene test. Spearman’s and Pearson’s analyses were conducted for correlation analysis in this study using R 4.0.2 and R Studio software. All statistical tests were two-sided, and a *P*-value < 0.05 was considered statistically significant.

## Results

### Periodically expressed transcripts during the cell cycle

**Fig. 2:**
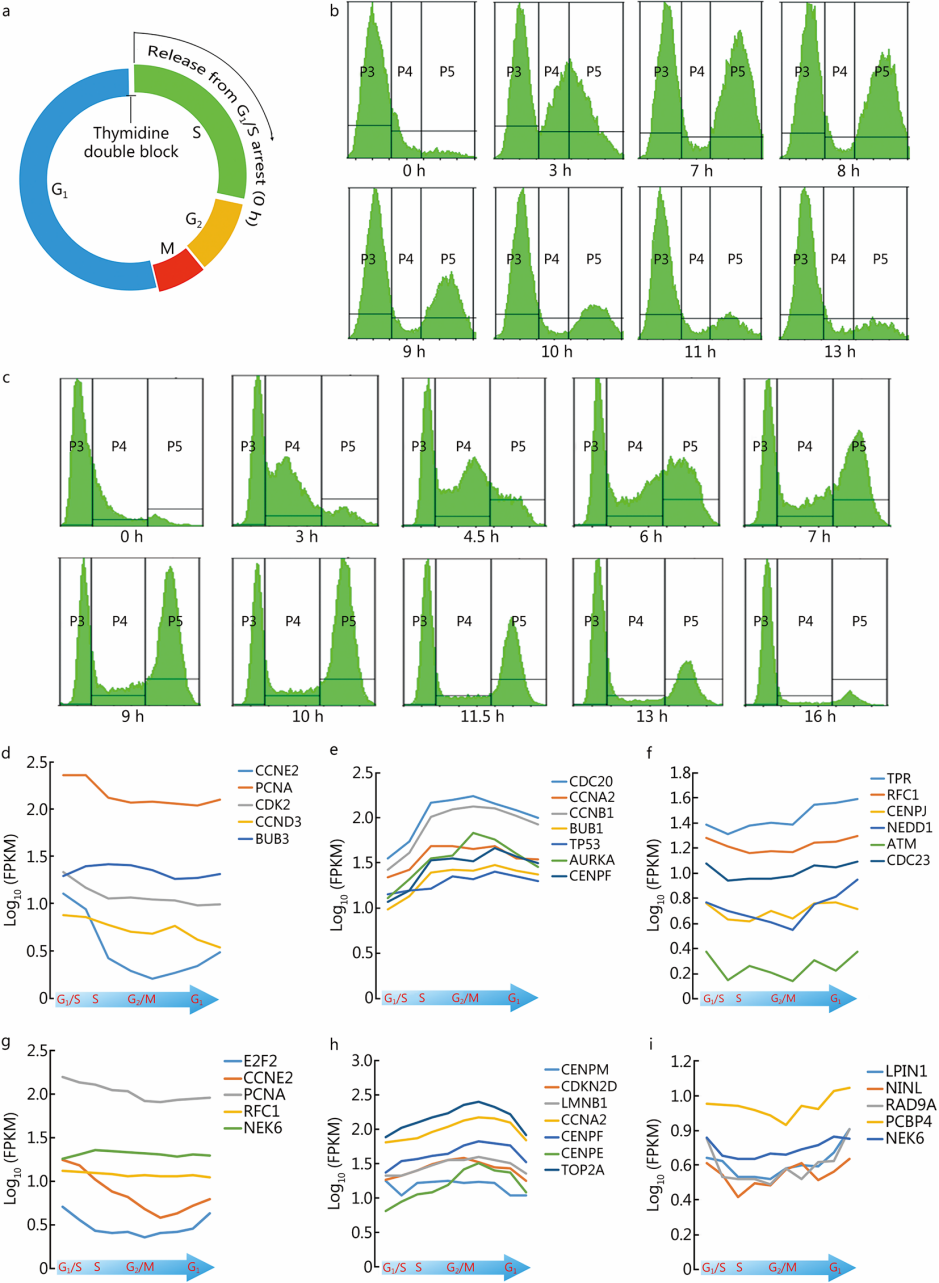
Establishment of cell cycle transcriptome datasets. **a** Time-point pattern of thymidine double block and cell cycle release. **b** Flow cytometry plots of the cell cycle distribution of synchronized HCT116 cells. P3/4/5 are the proportion of cells in G_1_, S, and G_2_/M phases, respectively. X-axis is the time since cell cycle release. **c** Flow cytometry plots of the cell cycle distribution of synchronized MDA-MB-231 cells. P3/4/5 are the proportion of cells in G_1_, S, and G_2_/M phases, respectively. X-axis is the time since cell cycle release. **d**–**f** FPKMs for some cell-cycle-related genes that are characteristically expressed in the G_1_/S, G_2_/M, and M-G_1_ phases in synchronized HCT116 cells, cells were harvested at 0, 3, 7, 8, 9, 10, 11, and 13 h after G_1_/S phase release, and diagram below indicates cell cycle stage. FPKM values of *CCNE2*, *PCNA*, *CDK2*, *CCND3*, and *BUB3* (**d**); FPKM values of *CDC20*, *CCNA2*, *CCNB1*, *BUB1*, *TP53*, *AURKA*, and *CENPF* (**e**); FPKM values of *TPR*, *RFC1*, *CENPJ*, *NEDD1*, *ATM*, and *CDC23* (**f**). **g**–**i** FPKMs for some representative genes that are highly expressed in the G_1_/S, G_2_/M, and M-G_1_ phases in MDA-MB-231 breast cancer cells, cells were harvested at 0, 3, 4.5, 6, 7, 9, 10, 11.5, 13, and 16 h after G_1_/S phase release, and diagram below indicates cell cycle stage. FPKM values of *E2F2*, *CCNE2*, *PCNA*, *RFC1*, and *NEK6* (**g**). FPKM values of *CENPM*, *CDKN2D*, *LMNB1*, *CCNA2*, *CENPF*, *CENPE*, and *TOP2A* (**h**); FPKM values of *LPIN1*, *NINL*, *RAD9A*, *PCBP4*, and *NEK6* (**i**). FPKM fragments per kilobase of exon model per million mapped fragments

Datasets derived from the analysis of transcriptome data during cell cycle progression in typical cancer cell models were retrieved, including data from cervical cancer HeLa, colon cancer HCT116 and breast cancer MDA-MB-231 cells. The high-quality and high-sequencing-depth HeLa cell sequential transcriptome dataset was published by Dominguez et al. [17] in 2016. Since algorithms available at that time could only detect a part of transcript splicing events compared with current algorithms, we adopted HISAT2 and StringTie to reanalyze the dataset. The HCT116 transcriptome was derived from GEO data previously published by our group [12]. For the current study, we also produced parallel in-depth sequential transcriptome data by cell cycle synchronization of MDA-MB-231 breast cancer cells. The cell cycle phases of HCT116 and MDA-MB-231 cells were successfully synchronized, and the cells were assessed by flow cytometry analysis (Fig. 2a–c). After the transcriptome was assessed, we extracted and analyzed the expression patterns of some typical cell cycle-related genes in HCT116 and MDA-MB-231 cells, including the genes expressed in the G_1_/S phases, such as *CCNE2*, *PCNA*, *CDK2*, *CCND3*, *BUB3*, *E2F2*, *RFC1* and *NEK6*; genes expressed in the G_2_/M phases, such as *CDC20*, *CCNA2*, *CCNB1*, *BUB1*, *TP53*, *AURKA*, *CENPF*, *CENPM*, *CDKN2D*, *LMNB1*, *CENPE* and *TOP2A*; and genes expressed in the M-G_1_ phases, such as *TPR*, *RFC1*, *CENPJ*, *NEDD1*, *ATM*, *CDC23*, *LPIN1*, *NINL*, *RAD9A*, *PCBP4* and *NEK6*. The results confirmed successful synchronization of the G_1_, S, G_2_ and M phases (Fig. 2d–i).

**Fig. 3:**
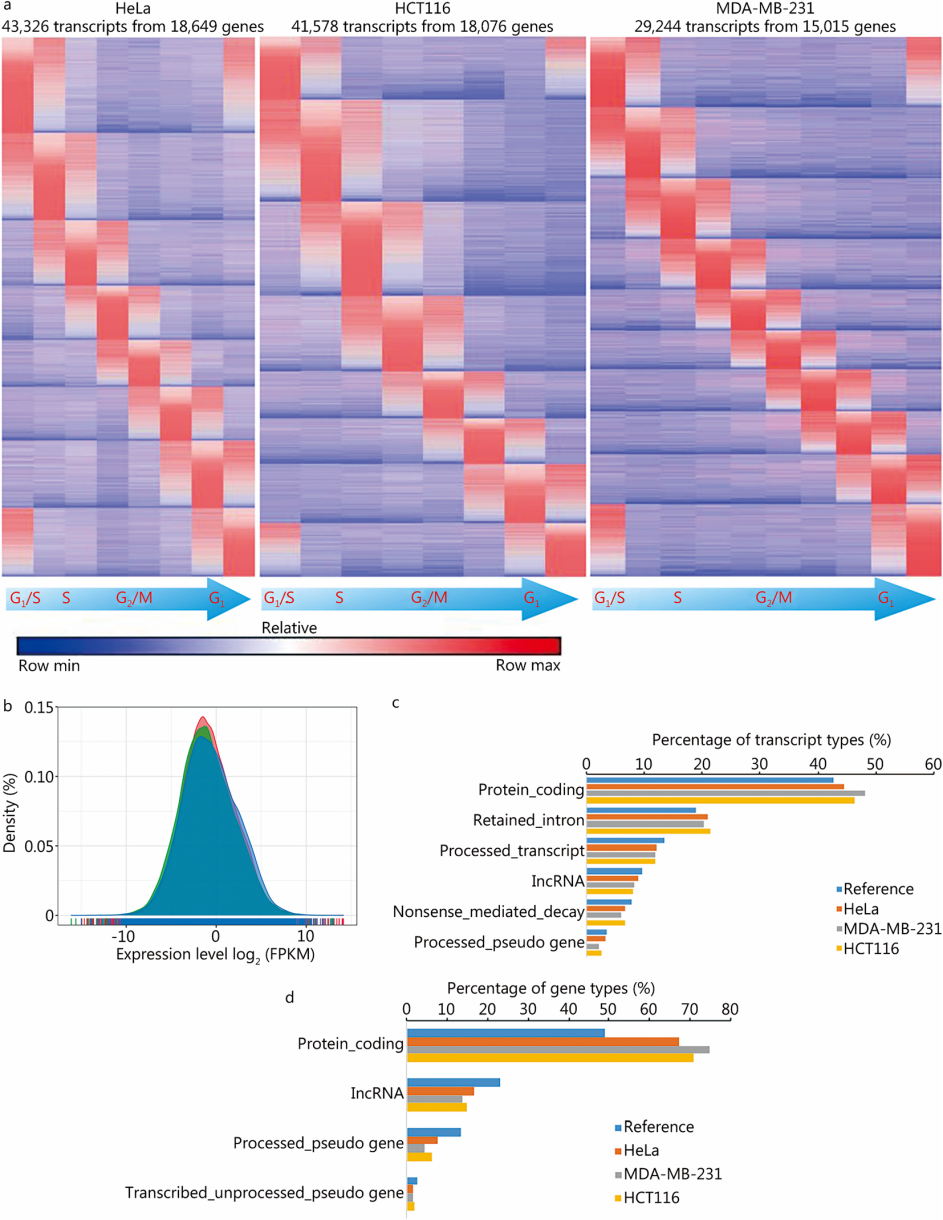
Expression patterns and characteristics of periodic transcripts in HeLa cervical cancer cells, HCT116 colon cancer cells and MDA-MB-231 breast cancer cells. **a** The rows show the normalized FPKM values of periodic transcripts to visualize their dynamic changes during the cell cycle. Each column represents a cell sample. They are samples at consecutive time points after cell cycle synchronization in G_1_/S phase. From left to right are samples in each phase of G_1_/S-S-G_2_/M-G_1_. Each row represents a transcript, and the transcripts from top to bottom are the transcripts highly expressed in each phase of G_1_/S-S-G_2_/M-G_1_. **b** Overall distribution of FPKM values for all the periodic transcripts in HeLa, HCT116 and MDA-MB-231 cells. **c** Bar plot shows the proportion of major types of periodic transcripts (the proportion is more than 1%). The reference is the collection of all expressed periodic and aperiodic transcripts to compare the enrichment of transcript types, the types of periodic transcripts in the figure are not significantly enriched. **d** Bar plot shows the proportion of the main types of genes periodic transcripts belong to (the proportion is more than 1%), and the reference is the collection of all expressed genes. Among the protein-coding gene type, the proportion of genes to which periodic transcripts belong is higher than that of the reference, indicating that the periodic transcripts were enriched to protein-coding genes. FPKM fragments per kilobase of exon model per million mapped fragments

To identify the transcripts that were periodically regulated during the cell cycle, we constructed an algorithm, applied it to the transcriptome dataset and identified the peak of each periodic transcript fluctuating with the cell cycle (see the Methods section). Compared with previously published algorithms [3, 17], our algorithm does not use classical periodic genes as references but it is dependent upon whether a gene has an expression peak during the cell cycle. This strategy greatly improves the sensitivity of the algorithm. Using this algorithm, we analyzed the transcriptome data of HeLa, HCT116 and MDA-MB-231 cells, and 43,326, 41,578 and 29,244 periodically expressed transcripts were identified. According to the position of the expression peak of each candidate transcript, we visualized the transcript expression as FPKM values throughout the G_1_, S, G_2_, and M phases in the three cancer cell models (Fig. 3a) and showed the expression pattern of the periodic transcripts across a complete cell cycle, the full transcriptome data are shown in the Additional file 1: Tables S1–S3. To understand the overall characteristic fluctuation of these transcripts, we further analyzed the average expression levels of each transcript in the panel of cell lines (Fig. 3b). We found that the overall levels of the periodic transcripts in the three cell types were similar. In addition, the transcript annotation categories in the three cell lines were also similar, with protein-coding transcripts accounting for more than 45% of the total transcripts (Fig. 3c). Furthermore, most of the corresponding genes of the transcripts (almost 70%) were protein-coding genes (Fig. 3d). These results suggest that most of the periodic transcripts were protein-coding and are thus capable of being targeted by current drug development strategies.

### A common set of cell cycle-related periodic transcripts in different cell lines

**Fig. 4:**
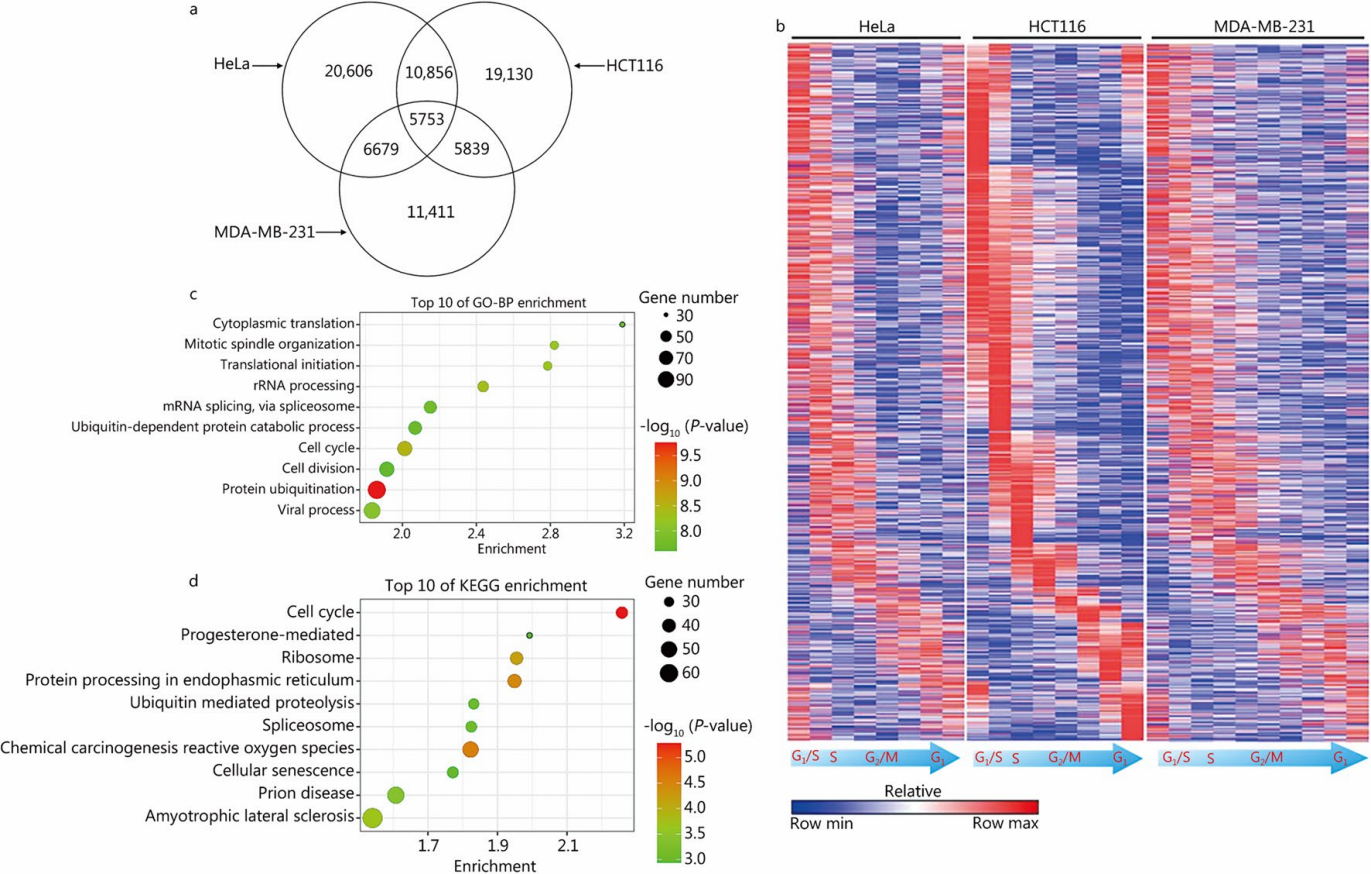
Functional enrichment of the common periodic transcripts. **a** Venn diagram showing the common periodic transcripts of HeLa, HCT116 and MDA-MB-231 cells. **b** Heatmap showing the 1280 transcripts with the same periodic expression patterns in HeLa, HCT116 and MDA-MB-231 cells. Each column represents a cell sample. They are samples at consecutive time points after cell cycle synchronization in G_1_/S phase. From left to right are samples in each phase of G_1_/S-S-G_2_/M-G_1_. Each row represents a transcript, and the transcripts from top to bottom are the transcripts highly expressed in each phase of G_1_/S-S-G_2_/M-G_1_. The heatmap is sorted according to the cell cycle phase of highly expressed transcripts in HCT116 cells, and the transcript order of HeLa and MDA-MB-231 is consistent with that of HCT116. **c**, **d** Bubble plots showing the top 10 GO-BP and KEGG pathway terms of the 1223 genes corresponding to the 1280 periodic transcripts. GO-BP Gene Ontology Biological Process, KEGG Kyoto Encyclopedia of Genes and Genomes

During cell division, a large number of genes may undergo systematic switching in their splicing patterns, which results in vastly diverse profiles of periodic transcripts. To extract the core sets of transcripts responsible for cell cycle regulation, we obtained the common periodic transcripts from the three cell lines (Fig. 4a). Among the 5753 transcripts shared in the three cell lines, there were 1280 transcripts whose expression peaks appeared in the same or adjacent cell cycle phases; that is, they showed restricted parallel expression. For these 1280 transcripts, we sorted their peak positions in HCT116 cells according to their cell cycle phases and plotted their pattern in all samples of HeLa, HCT116 and MDA-MB-231 cells (Fig. 4b). The full data are shown in the Additional file 1: Table S4. Furthermore, we performed GO-BP clustering analysis of the 1223 genes to which the 1280 transcripts corresponded and found that the main functions of the genes were related to the cell cycle, splicing and translation (Fig. 4c), and similar pathways were also significantly enriched in the KEGG analysis (Fig. 4d). These results suggest that most of the identified genes have been identified as cell cycle-related genes in previous studies, which further confirms the credibility of our analysis. Furthermore, the fact that the genes were enriched in RNA splicing functions indicates the importance of alternative splicing regulation in the cell cycle.

### Transcripts that correlate with drug sensitivity more stronger than drug-target genes

**Fig. 5:**
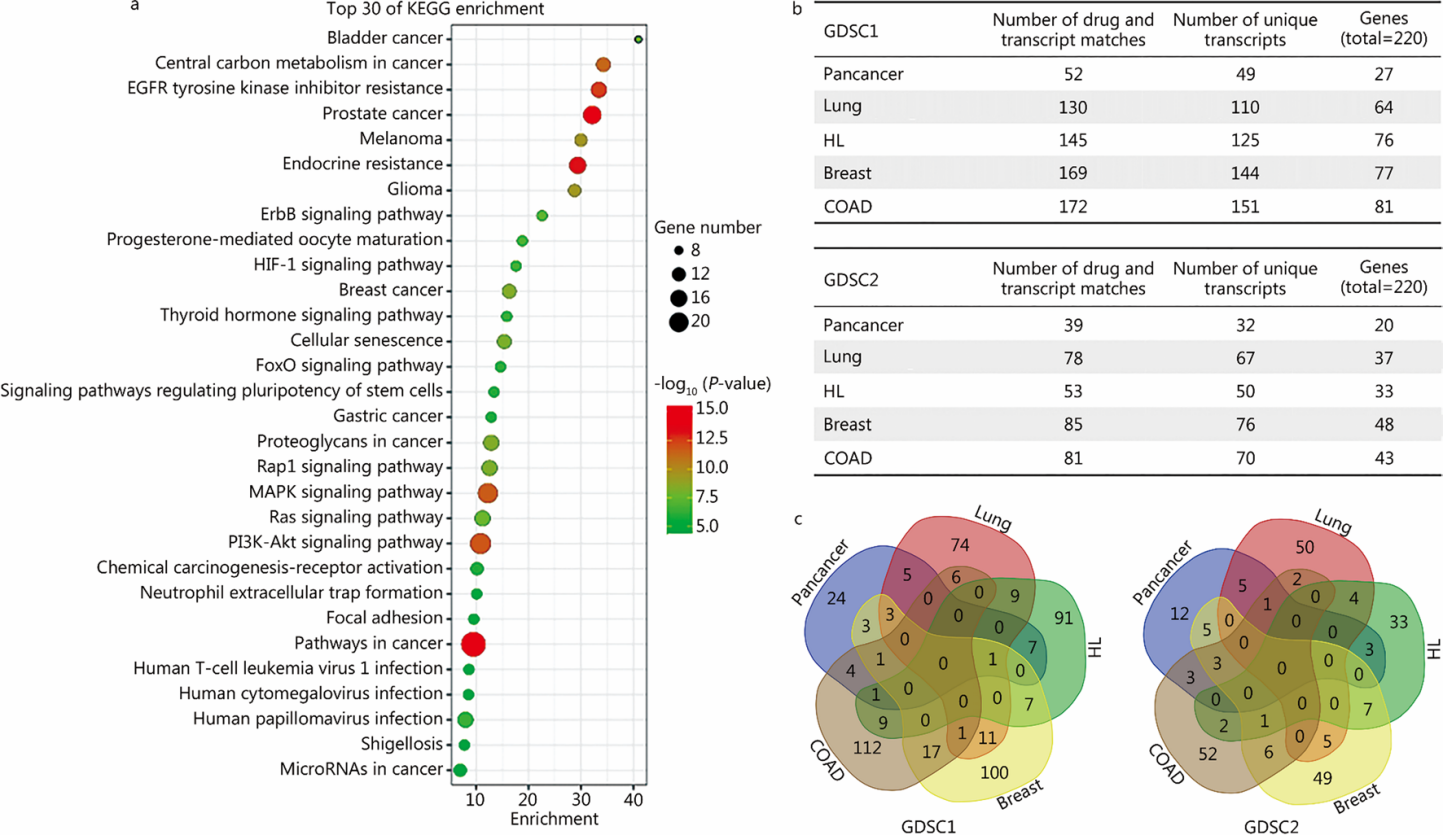
Functional and tissue-specific characteristics of drug sensitivity-related transcripts. **a** Bubble plot showing the cell signaling pathways of the drug sensitivity-related transcripts identified by KEGG enrichment analysis. **b** The number of drug sensitivity-related transcripts identified in different tumor types. **c** Venn diagram showing the distribution of drug sensitivity-related transcripts in different tumor types. KEGG Kyoto Encyclopedia of Genes and Genomes, HIF-1 hypoxia inducible factor-1, Fox forkhead box, COAD colon adenocarcinoma, HL hematopoietic and lymphoid tissue, GDSC Genomics of Drug Sensitivity in Cancer

To identify the cell cycle-related alternatively spliced transcripts, in addition to constructing transcriptome datasets of samples from different phases of the cell cycle, an integrated analysis of the relationship between cell cycle-related drugs and cell transcriptomes in large databases is another feasible solution. The two schemes can complement each other to further evaluate the potential clinical application of cell cycle-related transcripts. An accurate understanding of transcript expression patterns and their relationship with drug sensitivity is important for drug design and clinical application and optimization [20, 21]. Many modern tumor therapies target cell cycle checkpoints [22]. Whether cell cycle-based periodic transcripts have an important role in the regulation of drug sensitivity is a notable topic for investigation, and a better understanding of the relationship between periodic transcript expression and drug sensitivity can provide in-depth information regarding this matter. Given the lack of studies at the transcript level, we compared the correlation between anticancer drug sensitivity and the expression of targets at the gene or transcript level. The GDSC database was employed to extract the IC_50_ values of more than 400 drugs tested in a large panel of cancer cell lines [23]. The CCLE database includes deep transcriptomic sequencing data for thousands of cell types [24]. To efficiently obtain information from these databases, we developed a pipeline to calculate the correlation of drug sensitivity with transcripts targetable by drugs and the corresponding genes targetable by drugs, and we specifically focused on the transcripts that were more correlated with drug sensitivity than their corresponding genes. The transcript lists are shown in Additional file 1: Tables S5 and S6. The results revealed a large panel of transcripts that had higher correlations with drug sensitivity than their corresponding genes. KEGG clustering analysis of the corresponding genes indicated that the associated enrichment terms were related to broad functions and not to a specific biological process, for example, splicing-related functions (Fig. 5a). These results showed the complexity of drug sensitivity regulation beyond the intended target.

### Accurately assessing drug sensitivity by transcript levels, not gene levels

Since the pattern of transcript expression is highly tissue-specific and cancer type dependent, we carried out independent analyses based on cancer type to determine whether the relationship of transcript level with drug sensitivity is cancer type dependent. The total number of transcript-drug sensitivity correlation events and the number of involved unique transcripts are shown (Fig. 5b). The statistics of 220 drug-targeted genes showed that their transcripts were distributed extensively in each cancer type (Fig. 5b). Intersection analysis illustrated vastly different patterns of drug sensitivity-related transcripts in different types of tumors (Fig. 5c), which suggested that tissue specificity should be accounted for in transcript-based drug sensitivity analysis. The results in each cancer type also showed that there was a large group of transcript isoforms that had higher correlations with drug sensitivity than their corresponding genes, and the evaluation ability of these transcripts was more tissue-specific.

### Relationship between ENST00000547281 and increased resistance to CDK4/6 inhibitors

**Fig. 6:**
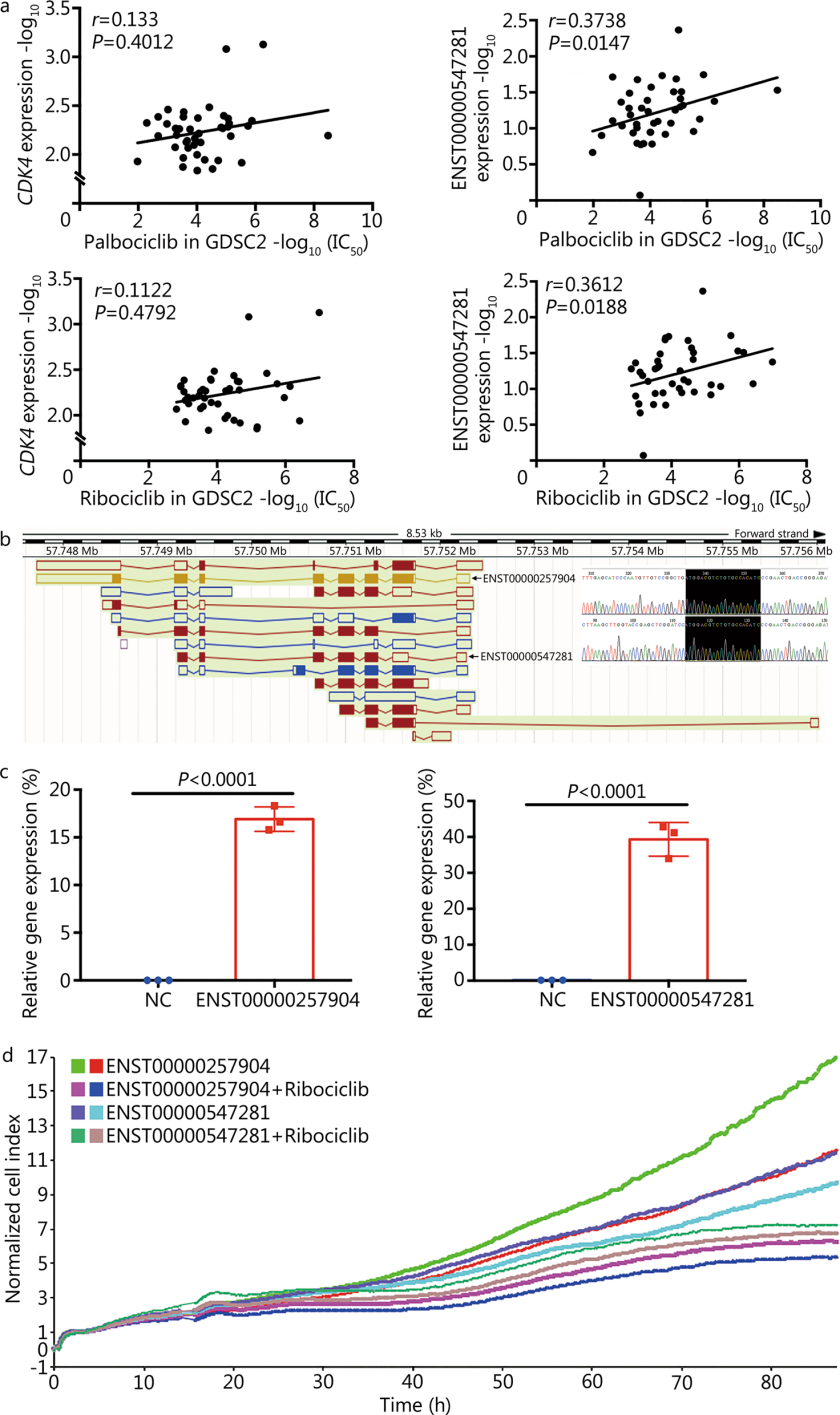
Relationship between ENST00000547281 and CDK4/6 inhibitor sensitivity. **a** Scatter diagrams of the palbociclib/ribociclib IC_50_ values and *CDK4* gene and CDK4 transcript ENST00000547281 levels. **b** Splicing pattern of all *CDK4* transcripts, the sequencing plot is part of the sanger sequencing of plasmid vectors carrying CDK4 transcripts ENST00000257904 and ENST00000547281, and the black box is the translation start site of ENST00000547281. In the structure plot and sequencing results, it can be seen that the sequence of ENST00000547281 transcript is part of ENST00000257904 transcript. **c** The histogram shows the relative expression levels of ENST00000257904 and ENST00000547281 by plasmid transfection into MCF7 cells, the left side is the ENST00000257904 plasmid group, and the right side is the ENST00000547281 plasmid group. **d** Line graph shows the proliferation and drug toxicity curves of MCF7 cells. The X-axis is the time after the cells were seeded into the plates, and the Y-axis is cell density index normalized by that at 0 h. NC negative control, GDSC Genomics of Drug Sensitivity in Cancer

CDK4, a target of palbociclib and ribociclib, is a major regulator of the G_1_/S phase transition [25]. According to the Ensembl database, compared with the CDK4 reference transcript ENST00000257904, the CDK4 alternative splicing transcript ENST00000547281 lacks exons 1, 2 and 7. We found that the correlations of sensitivity to the CDK4/6 inhibitors palbociclib and ribociclib with ENST00000547281 were significantly stronger than the correlations of sensitivity to the CDK4/6 inhibitors palbociclib and ribociclib with the *CDK4* gene in breast cancer (Fig. 6a). To confirm the biological significance of the correlations of these transcripts with sensitivity to CDK4/6 inhibitors, we constructed ENST00000257904 and ENST00000547281 transcript expression plasmids using the pCDNA3.1 vector (Fig. 6b), transfected the plasmids into MCF7 cells (a model of luminal A-type breast cancer, a subtype for which ribociclib treatment is used, Fig. 6c), and detected the sensitivity of the plasmid-transfected cells to ribociclib. The results showed that ENST00000547281 significantly increased the resistance of cells to ribociclib compared with ENST00000257904 based on growth inhibition after ribociclib treatment (Fig. 6d). These results further confirmed that the splicing and expression patterns of transcripts affect the correlations of transcripts with drug sensitivity.

### Mitosis-specific expression of splicing transcripts correlates with chemotherapy sensitivity

**Fig. 7:**
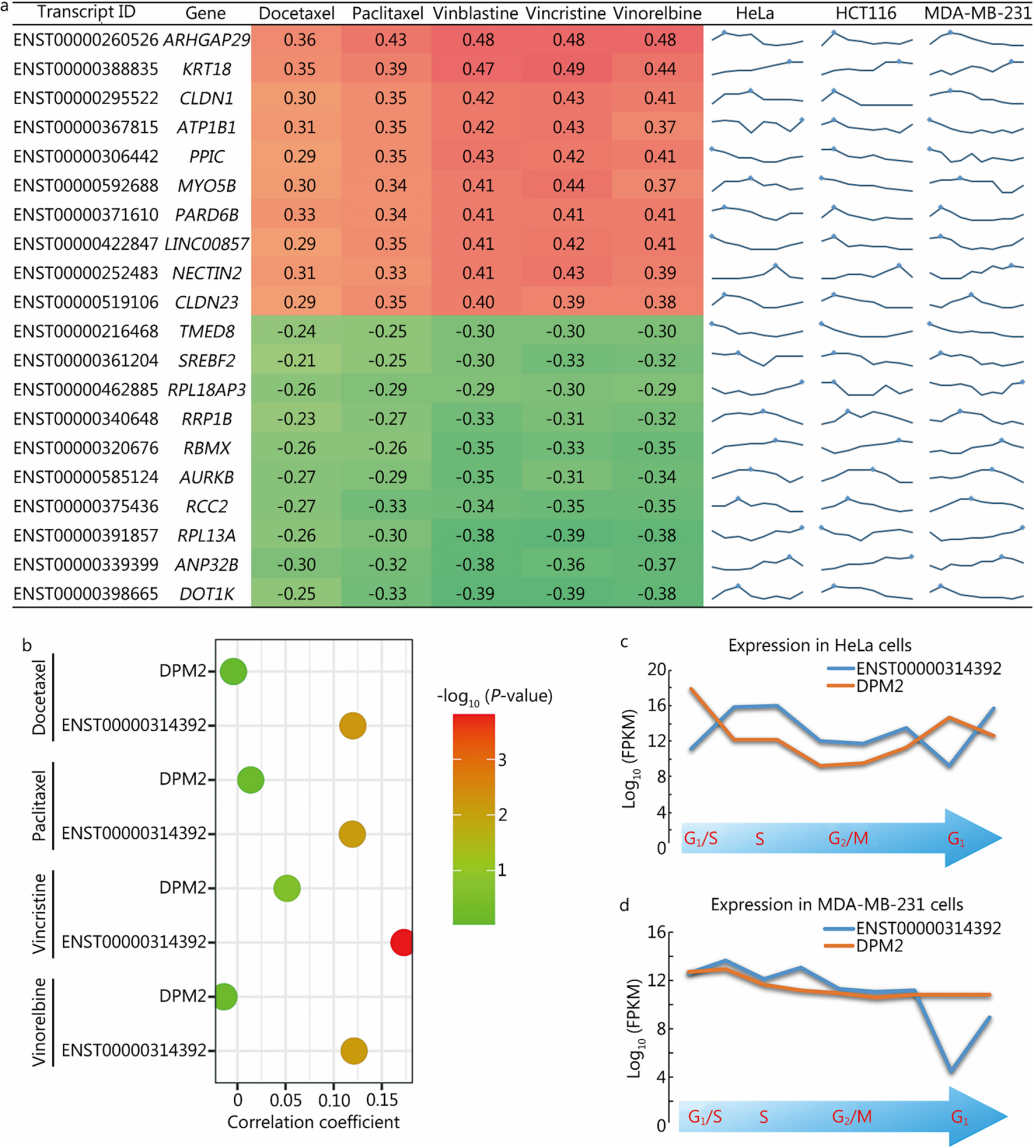
Relationship between periodic transcripts and chemotherapeutic drug sensitivity. **a** Heatmap listing the top 10 transcripts with the highest positive (ENST00000260526, ENST00000388835, ENST00000295522, ENST00000367815, ENST00000306442, ENST00000592688, ENST00000371610, ENST00000422847, ENST00000252483, and ENST00000519106) and negative (ENST00000216468, ENST00000361204, ENST00000462885, ENST00000340648, ENST00000320676, ENST00000585124, ENST00000375436, ENST00000391857, ENST00000339399, and ENST00000398665) correlations with sensitivity to docetaxel, paclitaxel, vinblastine, vincristine and vinorelbine. Line graphs showing the periodic expression patterns of the transcripts in HeLa, HCT116 and MDA-MB-231 cells are also shown. **b** Correlations of DPM2 and ENST00000314392 with sensitivity to docetaxel, paclitaxel, vincristine and vinorelbine. **c** Periodic expression of DPM2 and ENST00000314392 in HeLa cells. **d** Periodic expression of DPM2 and ENST00000314392 in MDA-MB-231 cells. ARHGAP29 rho-type GTPase-activating protein 29, KRT18 keratin 18, CLDN claudin, ATP1B1 ATPase Na^+^/K^+^ transporting subunit beta 1, PPIC peptidyl-prolyl cis–trans isomerase C, MYO5B myosin VB, PARD6B partitioning defective 6 homolog beta, LINC00857 long intergenic non-protein coding RNA 857, NECTIN2 nectin cell adhesion molecule 2, TMED8 transmembrane P24 trafficking protein family member 8, SREBF2 sterol regulatory element binding transcription factor 2, RPL18AP3 ribosomal protein L18a pseudogene 3, RRP1B ribosomal RNA processing 1B, RBMX RNA binding motif protein X-linked, AURKB aurora kinase B, RCC2 regulator of chromosome condensation 2, RPL13A ribosomal protein L13a, ANP32B acidic nuclear phosphoprotein 32 family member B, DOT1L DOT1 like histone lysine methyltransferase, DPM2 dolichyl-phosphate mannosyltransferase polypeptide 2

Mitotic arrest is an important mechanism used to kill cancer cells by antitumor drugs, including some currently widely used chemotherapeutic drugs and targeted drugs, such as paclitaxel and vinblastine [26]. A better understanding of the relationship between the newly identified periodic transcripts and drug sensitivity will facilitate the development of mitosis-related antitumor therapies. We further extracted the expression profiles of the periodic transcripts shown in Fig. 4a, from the CCLE transcriptome dataset analysis with relationships with sensitivity to mitosis- and cell cycle-related drug IC_50_ values in the different cell lines according to the GDSC database. The IC_50_ values for vinorelbine, paclitaxel and docetaxel in each cell type were extracted to calculate the correlation between transcript FPKM values and drug IC_50_ values in all cell types. The top 10 transcripts with positive (ENST00000260526, ENST00000388835, ENST00000295522, ENST00000367815, ENST00000306442, ENST00000592688, ENST00000371610, ENST00000422847, ENST00000252483, and ENST00000519106) and negative (ENST00000216468, ENST00000361204, ENST00000462885, ENST00000340648, ENST00000320676, ENST00000585124, ENST00000375436, ENST00000391857, ENST00000339399, and ENST00000398665) correlations are shown in Fig. 7a.

Using the drug sensitivity analysis pipeline described above, we obtained a set of periodic transcripts that outperformed their corresponding genes in drug sensitivity evaluation. For example, the ENST00000314392 transcript of DPM2 was significantly correlated with sensitivity to docetaxel, paclitaxel, vincristine and vinorelbine, which are cell cycle-related chemotherapeutic drugs (*P* < 0.05), and the correlations were significantly stronger than those of the corresponding *DPM2* gene (Fig. 7b). Further analysis using the periodic transcript expression data showed that in HeLa and MDA-MB-231 cells, the expression pattern of ENST00000314392 was significantly different from that of DPM2, with its expression level increasing during mitosis and decreasing after mitosis (Fig. 7c, d).

**Fig. 8:**
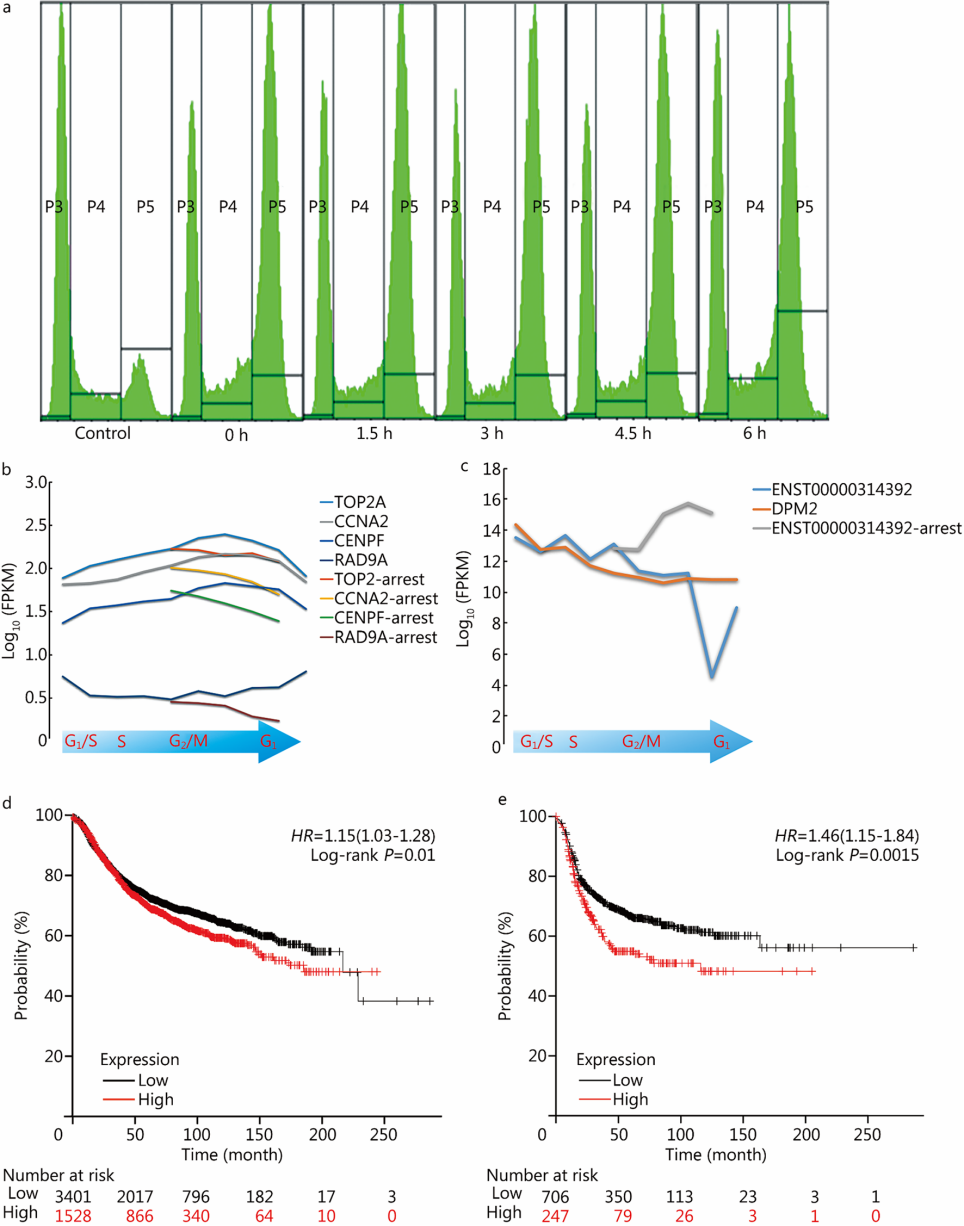
Specific alternative splicing of DPM2 during mitosis and its clinical relevance. **a** Flow cytometry panels showing the cell cycle distribution of MDA-MB-231 cells arrested with nocodazole and actinomycin D. The X-axis shows the time at which actinomycin D was added. **b** FPKM values of TOP2A, CCNA2, CENPF, and RAD9A under mitotic arrest. The long lines represent unarrested samples, and the short lines represent the samples with induced arrest by nocodazole and actinomycin D. The X-axis represents the cell cycle phases. **c** ENST00000314392 FPKM values under mitotic arrest and unblocked conditions. **d**, **e** RFS curves of DPM2 transcriptional expression in breast cancer and triple-negative breast cancer, respectively. FPKM fragments per kilobase of exon model per million mapped fragments, RFS relapse-free survival

To verify the results from the aforementioned comprehensive analysis of mitosis-related transcripts in different cell types, we chose MDA-MB-231 cells to perform further experiments. We subjected cycle-synchronized cells to nocodazole arrest after G_1_/S phase release and blocked RNA synthesis with actinomycin D when a majority of the cells began to enter M phase. Samples were collected at 0, 1.5, 3, 4.5 and 6 h after the addition of actinomycin D for transcriptome analysis. Quality control results showed that all the collected samples were stably maintained in the M phase, as shown by flow cytometry analysis (Fig. 8a), and the analysis of characteristic genes of cell division showed that their expression was stable and decreased gradually (Fig. 8b). Based on the results of the RNA-seq experiment, we found that the level of the ENST00000314392 transcript during mitosis was significantly different from that of DPM2, and the expression of ENST00000314392 increased significantly after cell division arrest (Fig. 8c). Since we inhibited transcription when we induced cell cycle mitosis phase arrest, the increased expression of ENST00000314392 was a result of alternative splicing regulation during cell division, which was consistent with the periodic transcript expression data. In addition, these results suggested that periodic transcripts can be validated by transcriptomic measurement after cell cycle arrest. The full transcriptome data are available under the GEO database (GSE216497).

As there was significant regulation of DPM2 gene alternative splicing in mitosis and ENST00000314392 showed a significant correlation with mitosis-related drug sensitivity, we believe that DPM2 may have a biological role in breast cancer progression. Survival analysis using the KMplot breast cancer dataset showed that high transcriptional expression of DPM2 was significantly correlated with poor relapse-free survival (RFS) (Fig. 8d), and in basal-like breast cancer, in which drugs such as paclitaxel and docetaxel are widely recommended by guidelines, high DPM2 transcriptional expression was significantly negatively correlated with poor RFS in the tumors of patients (Fig. 8e).

## Discussion

In this study, cell cycle phase models in three different cancer cell lines were established for deep transcriptome analysis, and an algorithm was developed to identify periodically expressed alternatively spliced transcripts in cells. Two previous important studies assessed periodic genes and alternative splicing events in HeLa cells using Fourier transform and Euclidean distance-based hierarchical clustering schemes, respectively. More than 1000 genes or alternative splicing events were characterized as being periodically expressed, and these studies laid the groundwork for large-scale screening of periodic genes and alternative splicing events [3, 17]. However, the algorithms used in the two studies less than 100 periodic expression events as references, which may have resulted in a large number of actual periodic events being missed. In contrast, identifying periodic expression events by using modified conditions and then comparing these periodic expression events in multiple cell types might reveal more periodic alternative splicing events. As such, we first developed an algorithm to optimize the efficiency of identifying splicing transcripts and their cell cycle-dependent fluctuations. The main idea of this algorithm was to identify peaks in an array by using numerical ranking without reference events. This algorithm had greater fault tolerance for waveforms to obtain more transcripts with periodic characteristics.

Screening and identification of cell cycle-related transcripts are scientifically and technically difficult. Due to the limitations of cell cycle synchronization and transcriptome-related technology, such analyses have only partially been accomplished [9, 27]. Research on the function of periodic transcripts in the cell cycle in cancer cells is also important. To clarify such functions, a global and precise understanding of the dynamic expression of transcripts during the cell cycle has to be achieved, which will be valuable for distinguishing potential functional variations of the proteins translated from transcript variants of the same gene, as well as their significance in cell cycle regulation and cancer treatment.

When we investigated the utility of the periodic transcripts in drug sensitivity evaluation, we first analyzed the utility of the corresponding genes in predicting  the sensitivity to various targeted drugs and found that a large number of transcript isoforms had a stronger correlation with drug sensitivity than their corresponding genes. We analyzed the structures of some of the transcripts and found that some contain targeting domains of the drugs, while many of them do not. Some studies have found that splicing-regulated protein isoforms are closely related to drug evaluation [28, 29], but more functions of the transcripts remain to be further explored.

In addition, we found that ribosome-related processes were major function in which the periodic transcripts were enriched. Whether this implies that systemic RNA alternative splicing occurs in a particular cell cycle phase-dependent manner (e.g., during nuclear membrane rupture during mitosis, resulting in changes in the liquid-phase environment of related components such as splicing proteins) or that ribosomal function changes to accommodate systemic gene splicing (e.g., translation processes are shut down by splicing ribosome-associated RNAs) needs to be explored in further research [30, 31].

## Conclusions

This study identified a panel of cell cycle-related periodic transcripts by assessing high-resolution phase-lapsed cell-cycle-synchronized transcriptomes. The host parental genes of most of the identified period transcripts are involved in cell cycle regulation, RNA splicing, and translation-related processes. We also found that the levels of transcripts of drug target genes have different values in evaluating drug sensitivity. We identified a series of transcripts exemplified by ENST00000547281 and ENST00000314392 for the potential role in drug sensitivity assessment. Overall, this study increases our understanding of the mechanism underlying fine cell cycle regulation, shows new directions for the application and exploration of cell cycle-targeted tumor therapy, and provides novel insights into alternative splicing-related drug development and evaluation.

## Supplementary Information

**Additional file 1: Table S1.** 43,326 periodic transcripts in HeLa cells. **Table S2.** 41,578 periodic transcripts in HCT116 cells. **Table S3.** 29,244 periodic transcripts in MDA-MB-231 cells. **Table S4.** 1280 periodic transcripts in the overlap of HeLa, HCT116, and MDA-MB-231 cells. **Table S5.** Correlations between transcript/corresponding gene FPKM values and the IC_50_ values of drugs in the GDSC1 dataset (the listed transcripts are those with correlations at least 0.1 higher or lower than those of their corresponding genes). **Table S6.** Correlations between transcript/host gene FPKM values and drug IC_50_ values in the GDSC2 dataset (the listed transcripts are those with correlations at least 0.1 higher or lower than those of their corresponding genes).

## Data Availability

The data obtained in the analysis of this article are included in this paper, and the RNA-seq raw data reported here are available under GEO series GSE81485, GSE123959 and GSE216497. All the drug IC_50_ data of the cell lines were downloaded from the GDSC database. The IC_50_ data from GDSC1 and GDSC2 were analyzed separately. The drug target gene information was mainly derived from the GDSC database. Multitarget drugs were excluded from our analysis to avoid misinterpretation. Cell lines without target drug evaluations in the database were deleted (https://www.cancerrxgene.org/). Transcriptomic expression data of all the cell lines were downloaded from the CCLE database. CCLE cell lines were matched with the GDSC1 and GDSC2 drug IC_50_ datasets. We focused on the genes and corresponding transcripts targeted by the drugs from both the GDSC1 and GDSC2 datasets (https://depmap.org/portal/). Gene and transcript annotation were performed with Ensembl Biomart [32], and the structural information of the transcripts was downloaded from the Ensembl Gene Browser (https://asia.ensembl.org/index.html).

## Abbreviations

ACVR1B: Activin A receptor type 1B
ANP32B: Acidic nuclear phosphoprotein 32 family member B
AKT: AKT serine/threonine kinase
ALK: ALK receptor tyrosine kinase
ARHGAP29: Rho-type GTPase-activating protein 29
ATP1B1: ATPase Na^+^/K^+^ transporting subunit beta 1
ATR: ATR serine/threonine kinase
AURKB: Aurora kinase B
BRD4: Bromodomain containing 4
BRDT: Bromodomain testis associated
CAPN1: Calpain 1
CCLE: Cancer cell line encyclopedia
CDK: Cyclin dependent kinase
CLDN: Claudin
COAD: Colon adenocarcinoma
CSF1R: Colony stimulating factor 1 receptor
DOT1L: DOT1 like histone lysine methyltransferase
DPM2: Dolichyl-phosphate mannosyltransferase polypeptide 2
EGFR: Epidermal growth factor receptor
EIF2A: Eukaryotic translation initiation factor 2A
EIF2AK3: Eukaryotic translation initiation factor 2 alpha kinase 3
ERBB2: Erb-B2 receptor tyrosine kinase 2
ESR1: Estrogen receptor 1
FGFR: Fibroblast growth factor receptor
Fox: Forkhead box
FPKM: Fragments per kilobase of exon model per million mapped fragments
GDSC: Genomics of Drug Sensitivity in Cancer
GO-BP: Gene Ontology Biological Process
HDAC: Histone deacetylase
HIF-1: Hypoxia inducible factor-1
HL: Hematopoietic and lymphoid tissue
HSP90AA1: Heat shock protein 90 alpha family class A member 1
IC_50_: 50% Inhibitory concentration
IGF1R: Insulin like growth factor 1 receptor
KEGG: Kyoto Encyclopedia of Genes and Genomes
KRT18: Keratin 18
LCK: Lymphocyte cell-specific protein-tyrosine kinase
LINC00857: Long intergenic non-protein coding RNA 857
MAP2K2: Mitogen-activated protein kinase kinase 2
MAPK: Mitogen-activated protein kinase
MDM2: Mouse double minute 2
MKNK2: MAPK interacting serine/threonine kinase 2
MRE11: Meiotic recombination 11 homolog 1
MTOR: Mechanistic target of rapamycin kinase
MYO5B: Myosin VB
NC: Negative control
NECTIN2: Nectin cell adhesion molecule 2
PARD6B: Partitioning defective 6 homolog beta
PDGFRB: Platelet derived growth factor receptor beta
PPIC: Peptidyl-prolyl cis–trans isomerase C
RBMX: RNA binding motif protein X-linked
RCC2: Regulator of chromosome condensation 2
RFS: Relapse-free survival
RPL13A: Ribosomal protein L13a
RPL18AP3: Ribosomal protein L18a pseudogene 3
RPS6KA3: Ribosomal protein S6 kinase A3
RRP1B: Ribosomal RNA processing 1B
RTCA: Real-time cell analyzer
SRC: Sarcoma
SREBF2: Sterol regulatory element binding transcription factor 2
STK10: Serine/threonine kinase 10
TMED8: Transmembrane P24 trafficking protein family member 8
